# Editorial: Insights into the diagnosis, pathogenesis and management of Takayasu arteritis

**DOI:** 10.3389/fimmu.2026.1896741

**Published:** 2026-06-12

**Authors:** Durga Prasanna Misra, Corrado Campochiaro, Fatma Alibaz-Oner, Alessandro Tomelleri

**Affiliations:** 1 Department of Clinical Immunology and Rheumatology, Sanjay Gandhi Postgraduate Institute of Medical Sciences (SGPGIMS), Lucknow, India; 2 Unit of Immunology, Rheumatology, Allergy and Rare Diseases, IRCCS San Raffaele Scientific Institute, Milan, Italy; 3 Division of Rheumatology, Department of Internal Medicine, School of Medicine, Marmara University, Istanbul, Türkiye

**Keywords:** arterial fibrosis, cardiovascular disease, genetics, glucocorticoid, mortality, Takayasu’s arteritis, tocilizumab (IL-6 inhibitor), ultrasound

This Research Topic includes articles on genetic risk factors, therapy with immunomodulatory cytokines, arterial wall remodeling, coronary artery involvement, cardiac surgery, and a novel ultrasound arterial imaging technique in patients with Takayasu arteritis (TAK).

Immune activation in the inflamed artery underlies the use of glucocorticoids as first-line treatment for active TAK (1). Peron Filho et al. report the association of polymorphisms in various genes related to glucocorticoid action and responsiveness in 81 patients with TAK. They identified that the 9β polymorphism in the *NR3C1* gene (linked with resistance to glucocorticoid therapy) was a risk factor for a greater number of adverse events with glucocorticoid therapy and also associated with ischemic events (multivariable-adjusted odds ratio 4.37, 95% CI 1.04-18.34). Given that cardiovascular events are the leading cause of mortality in patients with TAK, it is moot to consider testing for this polymorphism to stratify patients with TAK at greater risk of ischemic events and glucocorticoid resistance (2). In such patients, it might be prudent to consider early initiation of biologic agents such as tumor necrosis factor-alpha (TNF-α) inhibitors or interleukin-6 (IL-6) antagonism with tocilizumab for their glucocorticoid-sparing and potent immunosuppressive effects (1, 3). In another article, Hu et al. report the case of a young female with TAK initiated on tocilizumab (IL-6 receptor antagonist) for relapsing disease. Over seven years of treatment, recanalization and reversal of narrowing of multiple involved arteries in the aortic arch was observed. Arterial wall narrowing is a consequence of arterial injury resulting in fibrosis of the arterial wall. IL-6 is involved in both inflammatory responses and fibroblast activation. While reversal of arterial stenosis might be attributable to IL-6 inhibition, arterial wall remodeling also occurs continuously in TAK, even with a narrowed, fibrosed artery (4). Various matrix metalloproteinases (MMP- 1, 2, 3, 9, 13) and their inhibitors [tissue inhibitors of metalloproteinase (TIMP) 1 and 3], and other molecules such as CD147 and galectin-3 which induce extracellular metalloproteinases are involved in this process. Tocilizumab is widely used in TAK based on secondary outcomes of a randomized controlled trial (reduction in relapses on per-protocol but not the primary intention-to-treat analysis) and multiple observational studies (3). A few reports also suggest recanalization of affected arteries in TAK with other drugs such as the Janus kinase 1 inhibitor upadacitinib (5). In this context, another article by Espinosa-Bautista et al. reports epigenetic modulation of arterial wall remodeling with the long non-coding RNA (lncRNA) *HOX transcript antisense RNA* (*HOTAIR)* in patients with TAK. They observed lower *HOTAIR* expression in peripheral blood mononuclear cells (PBMCs) of TAK than age- and sex-similar healthy controls. A weak negative correlation was observed between *HOTAIR* expression on PBMCs and circulating MMP-2 and CD147. Recent literature suggests that extracellular matrix proteins drive arterial damage in TAK. Stojanovic and colleagues evaluated the association between the enhanced liver fibrosis (ELF) score and its components TIMP-1, procollagen 3 aminoterminal propeptide (PIIINP), and hyaluronic acid (HA) with arterial damage in patients with TAK. They reported a significant positive correlation between ELF score and clinically or angiographically-assessed damage scores. Further, PIIINP and HA were also significantly correlated with angiographically-assessed damage scores (6). In another recent paper, Qamar and colleagues reported longitudinal changes in circulating TIMP-1, PIIINP, HA, and the pro-fibrotic cytokine transforming growth factor-beta (TGF-β) in patients with TAK. During the transition from active to inactive disease, an increase in all these circulating markers of fibrosis was observed (7). These observations position arterial wall fibrosis as a leading therapeutic target for exploration in TAK.

As discussed earlier, cardiovascular disease is the leading cause of death in patients with TAK (2). Farrukh et al. reported coronary artery involvement in 13/137 (9%) of patients with TAK from a large single-center cohort from the National Institutes of Health, Bethesda, USA. An association between activity on 18-F fluorodeoxyglucose positron emission tomography and coronary arteritis was observed. The literature suggests similar outcomes with medical therapy or coronary artery revascularization in patients with TAK. Coronary artery bypass is associated with fewer subsequent cardiovascular events than percutaneous coronary intervention (8). In this study, 8/13 patients required open surgical bypass or percutaneous coronary artery intervention, with another two requiring repeated interventions. In another study, Liu et al. report outcomes in 64 patients with TAK undergoing cardiac surgery compared with 64 propensity score-matched non-TAK patients undergoing a similar procedure. Patients with TAK had similar risk of mortality or reoperation to non-TAK controls. However, a greater risk of hospitalization for cardiovascular causes and need for cardiac surgery for a new indication was observed in TAK. Despite clinically inactive disease at surgery, 8/64 patients had ongoing activity on arterial tissue histopathology. Clinicians should strive for disease quiescence during vascular procedures in patients with TAK for better surgical outcomes and survival (9).

Increasingly, ultrasound is being used for angiographic assessment of TAK. The Outcome Measures in Rheumatology (OMERACT) has recently proposed definitions of three elementary lesions, macaroni sign, stenosis, and occlusion, in patients with TAK (10). In this context, a scoping review by Soto-Fajardo et al. identifies the expanding literature on the use of contrast-enhanced ultrasound (CEUS) in TAK. CEUS, while technically challenging, reveals neovascularization of the inflamed arterial wall, which is associated with disease activity in patients with TAK. While the preliminary data are encouraging, CEUS needs further evaluation in patients with TAK before its use translates into clinical care.

Despite advances in the management of TAK presented in this Research Topic, several important unmet needs remain. Future research should focus on developing precision medicine approaches integrating genetic, molecular, and imaging biomarkers to improve risk stratification and guide treatment decisions. Better understanding of the mechanisms underlying arterial wall remodeling, fibrosis, and vascular repair might identify novel therapeutic targets to prevent irreversible vascular damage and improve long-term outcomes. In parallel, dedicated studies are needed to better characterize cardiovascular involvement, optimize the timing and outcomes of vascular interventions, and validate emerging imaging techniques, including CEUS. Ultimately, the integration of these advances might enable a more personalized approach to disease monitoring and treatment in patients with TAK.
